# Supplementary material for: Chronic Ouabain Targets Pore-Forming Claudin-2 and Ameliorates Radiation-Induced Damage to the Rat Intestinal Tissue Barrier
Source: Int J Mol Sci. 2023 Dec 24;25(1):278. doi: 10.3390/ijms25010278 (PMC10778734; doi:10.3390/ijms25010278)

Jejunum

Claudin-1

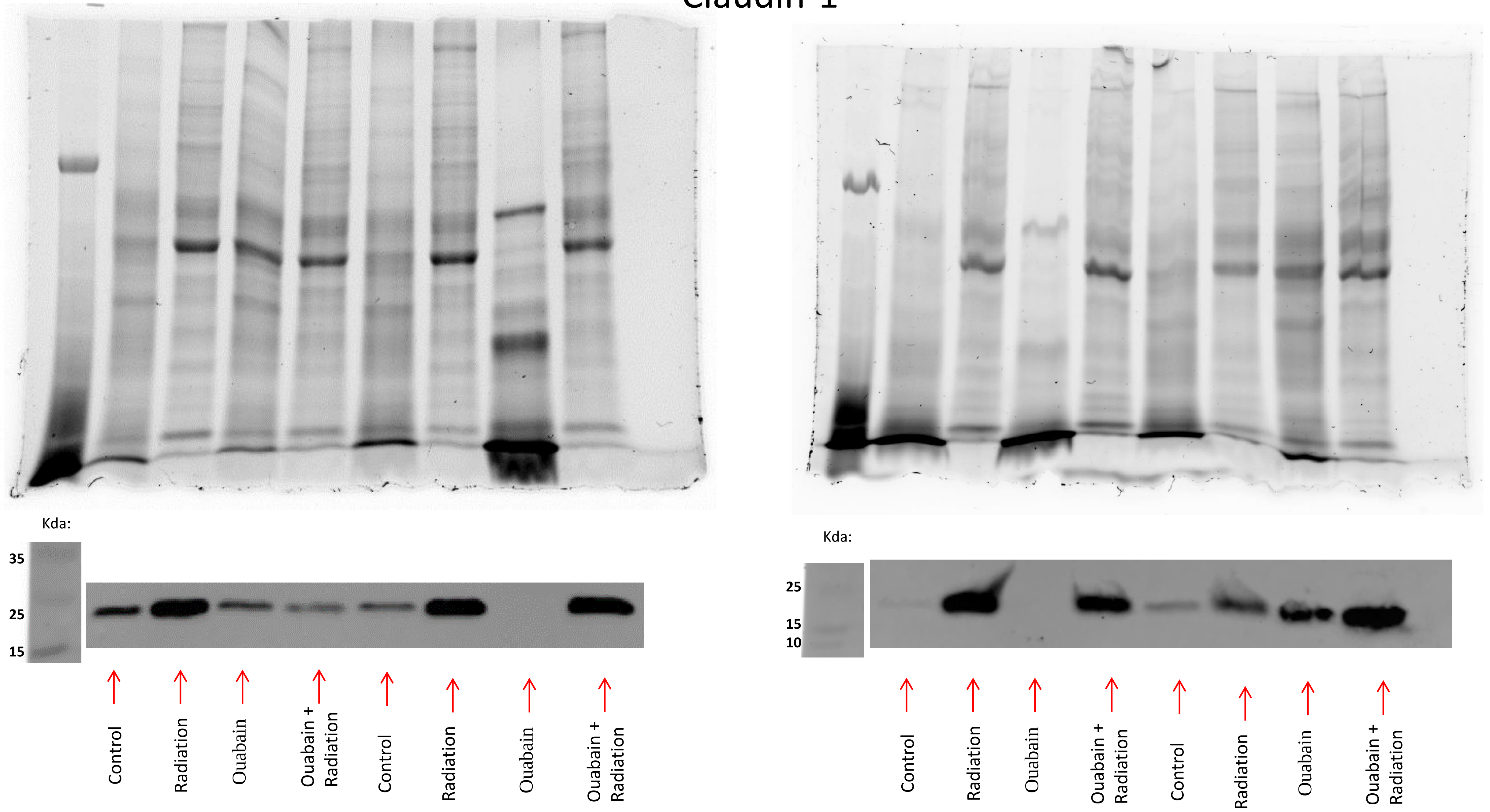
Kda:
35
25
15
Control
Radiation
Ouabain
Ouabain + Radiation
Control
Radiation
Ouabain
Ouabain + Radiation
Kda:
25
15
10
Control
Radiation
Ouabain
Ouabain + Radiation
Control
Radiation
Ouabain
Ouabain + Radiation

Claudin-2

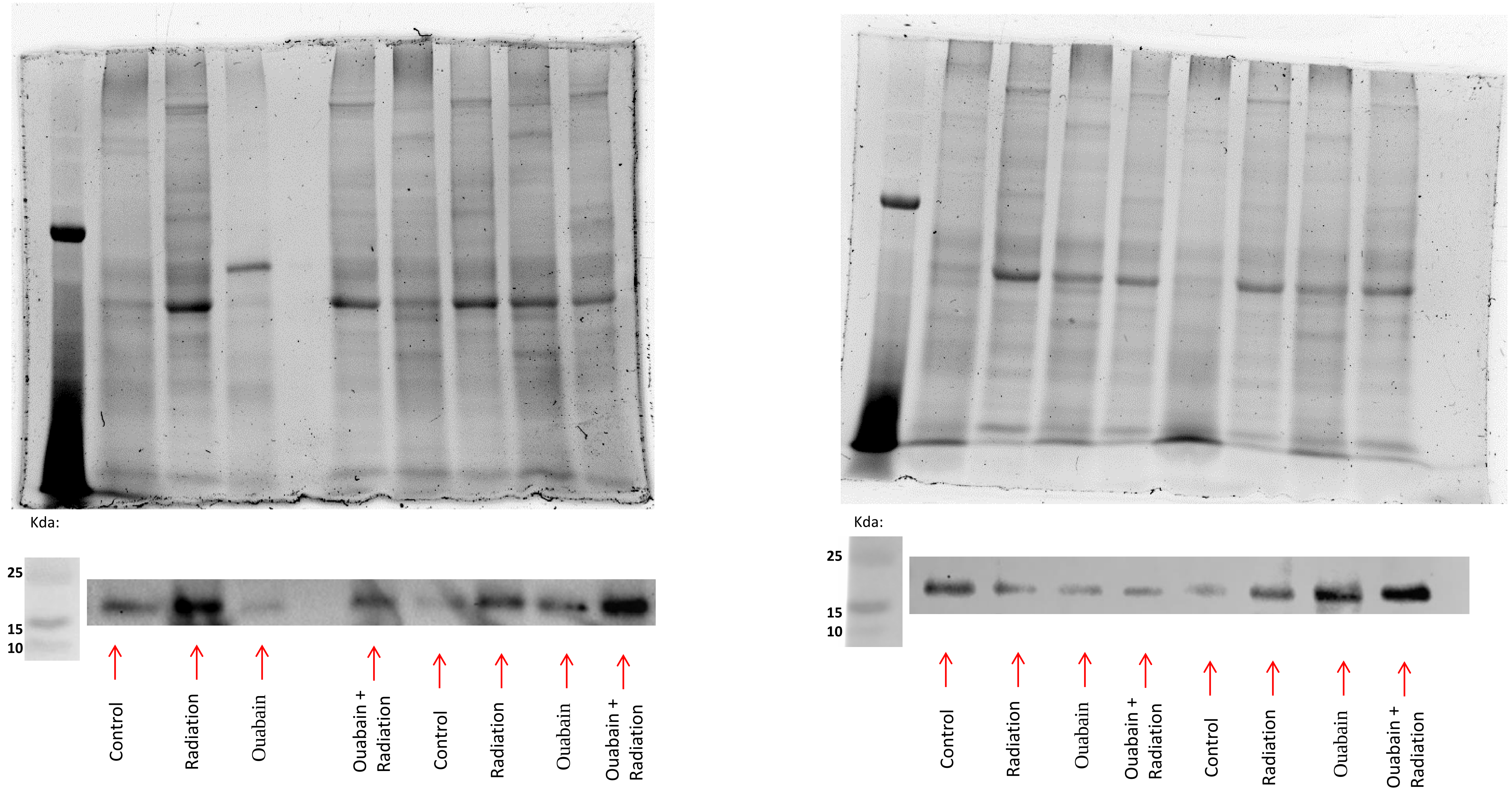
Kda:
25
15
10
Control
Radiation
Ouabain
Ouabain + Radiation
Control
Radiation
Ouabain
Ouabain + Radiation
Kda:
25
15
10
Control
Radiation
Ouabain
Ouabain + Radiation
Control
Radiation
Ouabain
Ouabain + Radiation

Claudin-3

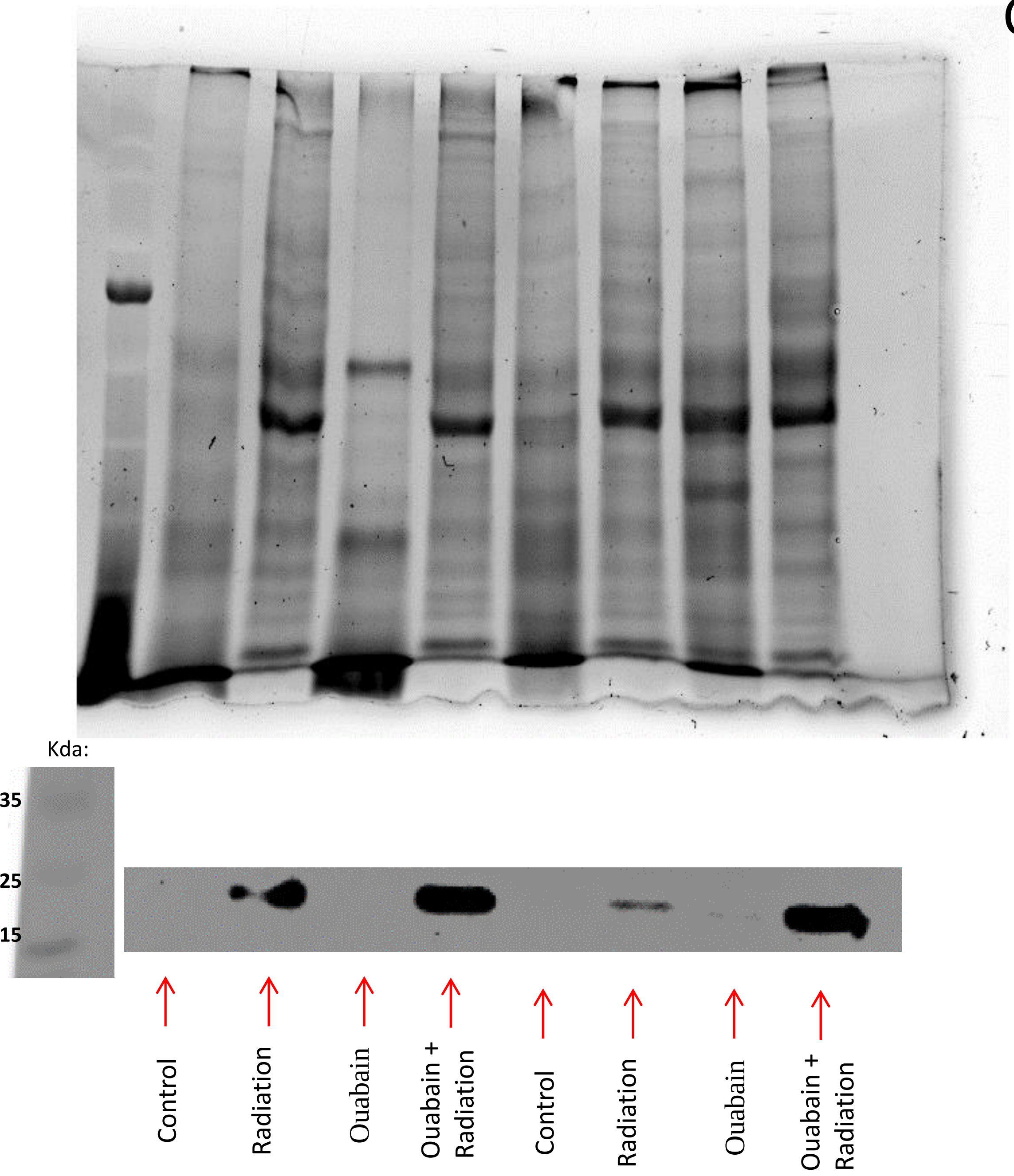
Kda:
35
25
15
Control
Radiation
Ouabain
Ouabain + Radiation
Control
Radiation
Ouabain
Ouabain + Radiation

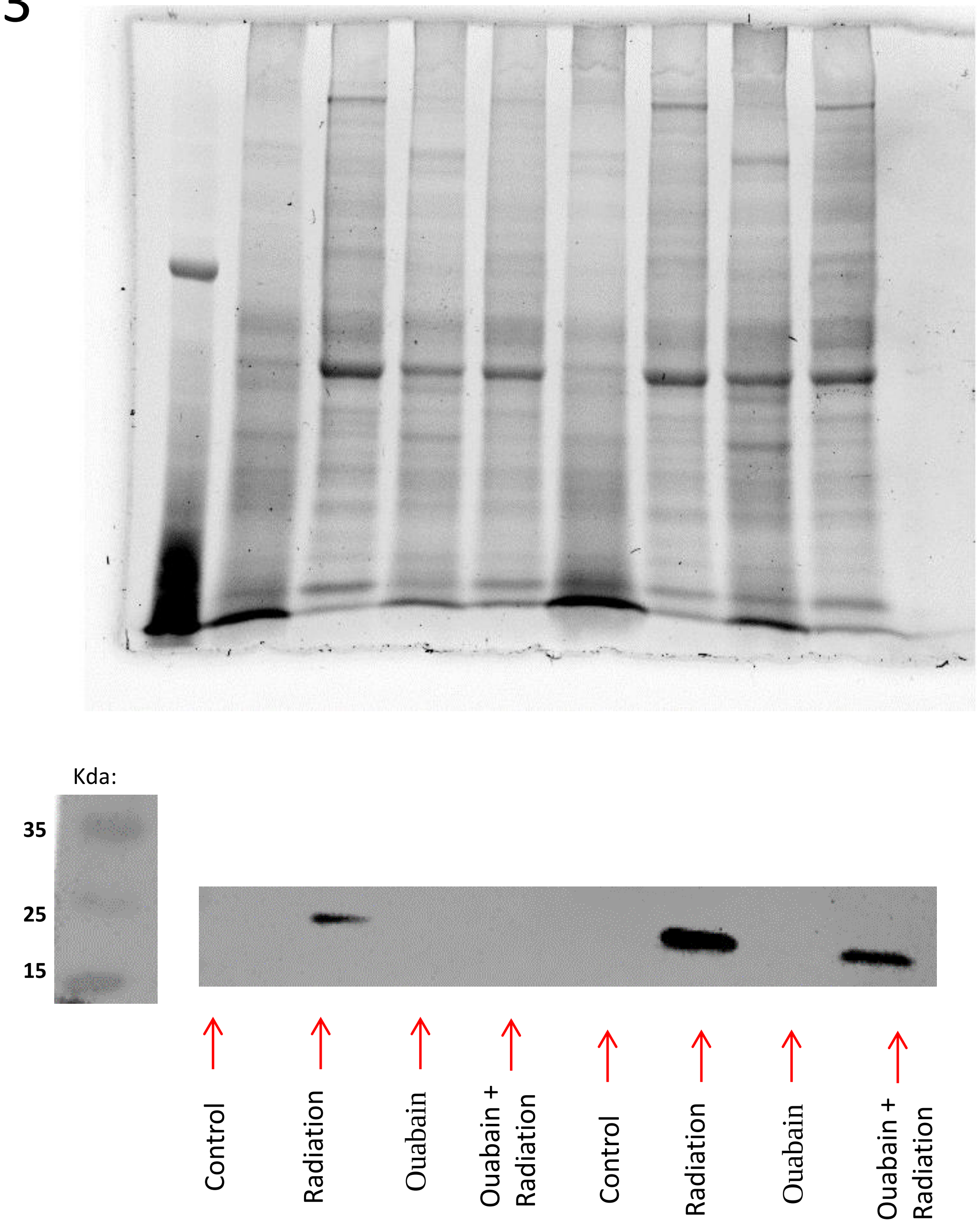
Kda:
35
25
15
Control
Radiation
Ouabain
Ouabain + Radiation
Control
Radiation
Ouabain
Ouabain + Radiation

Claudin-4

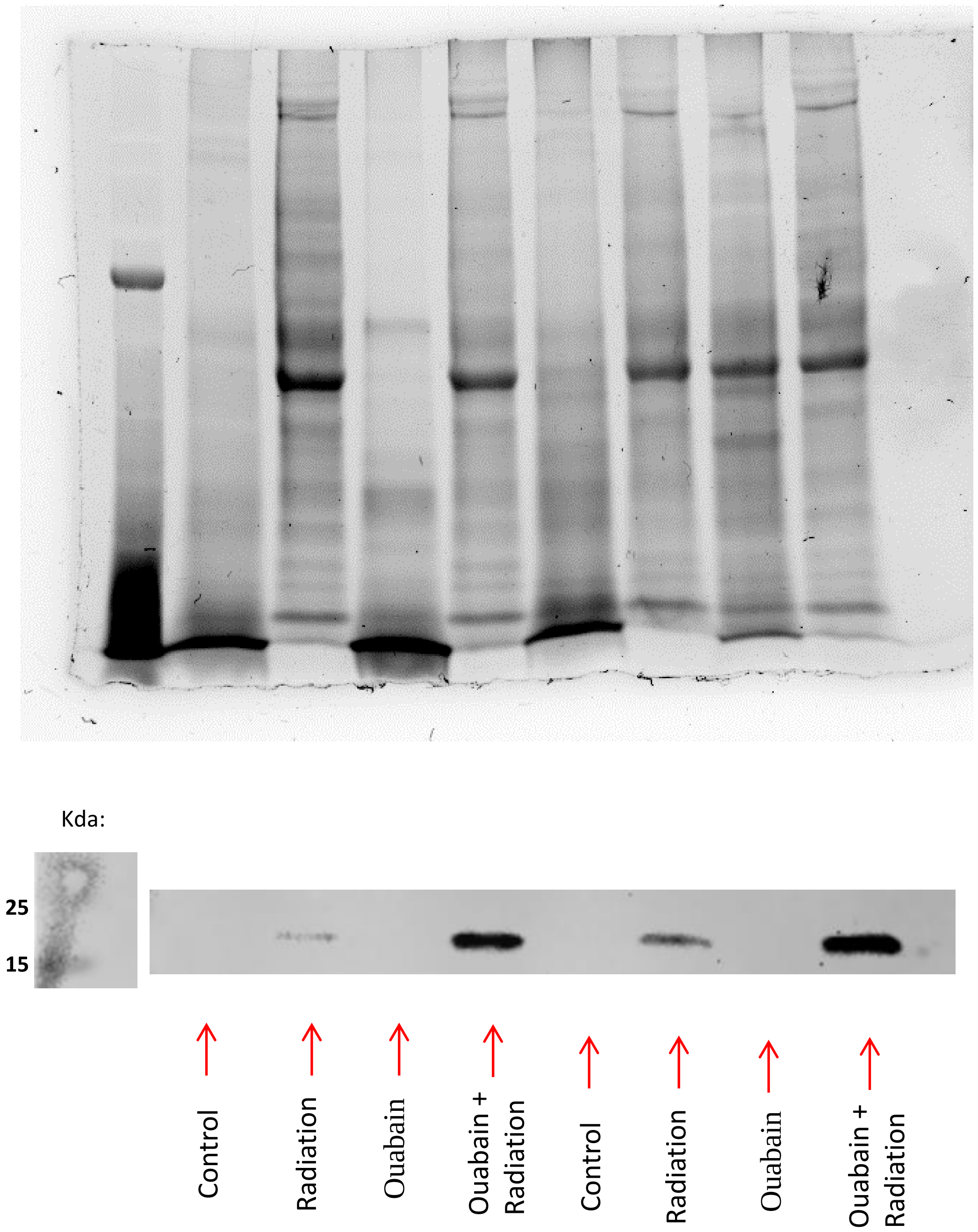
Kda:
25
15
Control
Radiation
Ouabain
Ouabain + Radiation
Control
Radiation
Ouabain
Ouabain + Radiation

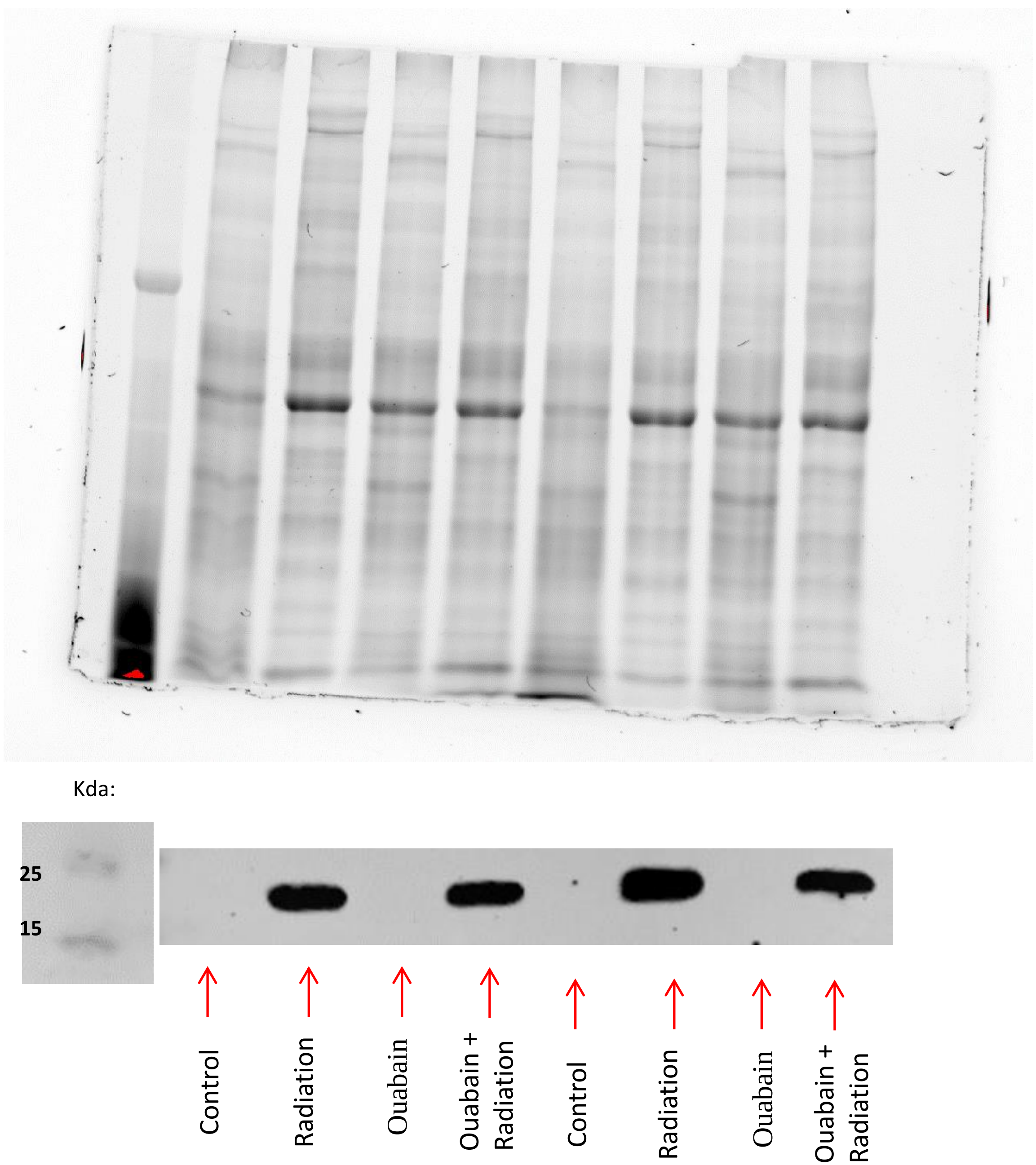
Kda:
25
15
Control
Radiation
Ouabain
Ouabain + Radiation
Control
Radiation
Ouabain
Ouabain + Radiation

Occludin

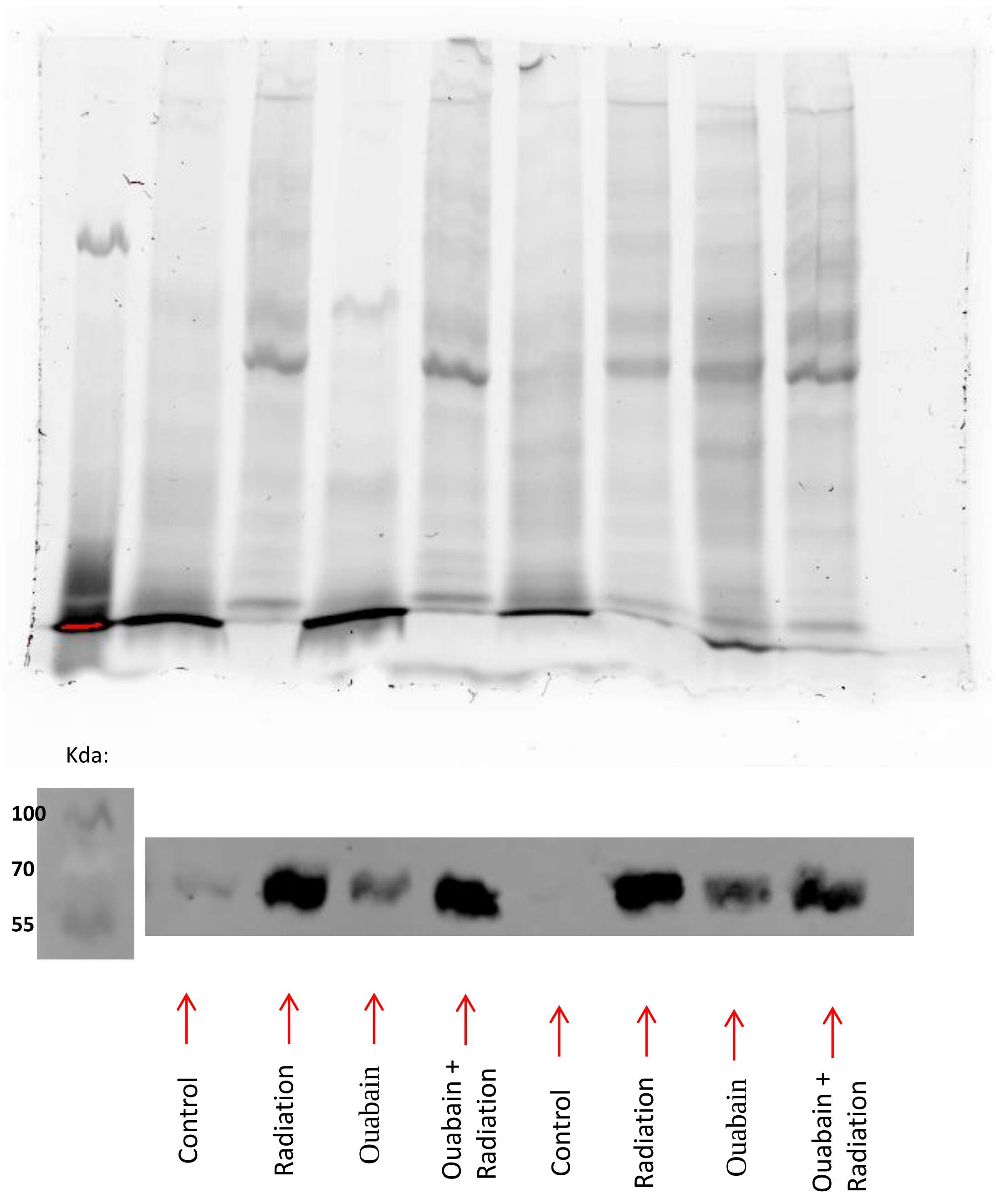
Kda:
100
70
55
Control
Radiation
Ouabain
Ouabain + Radiation
Control
Radiation
Ouabain
Ouabain + Radiation

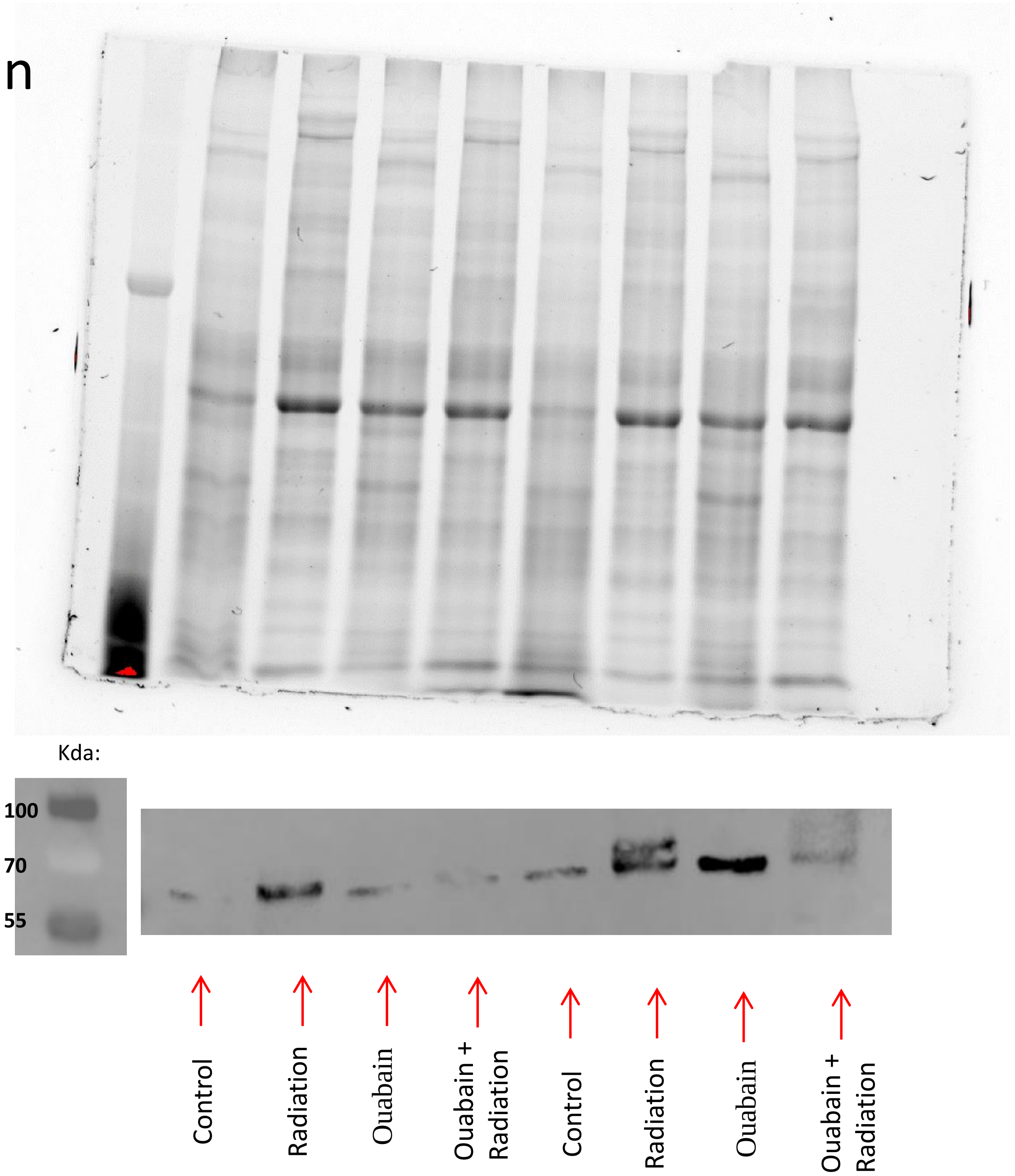
Kda:
100
70
55
Control
Radiation
Ouabain
Ouabain + Radiation
Control
Radiation
Ouabain
Ouabain + Radiation

Tricellulin

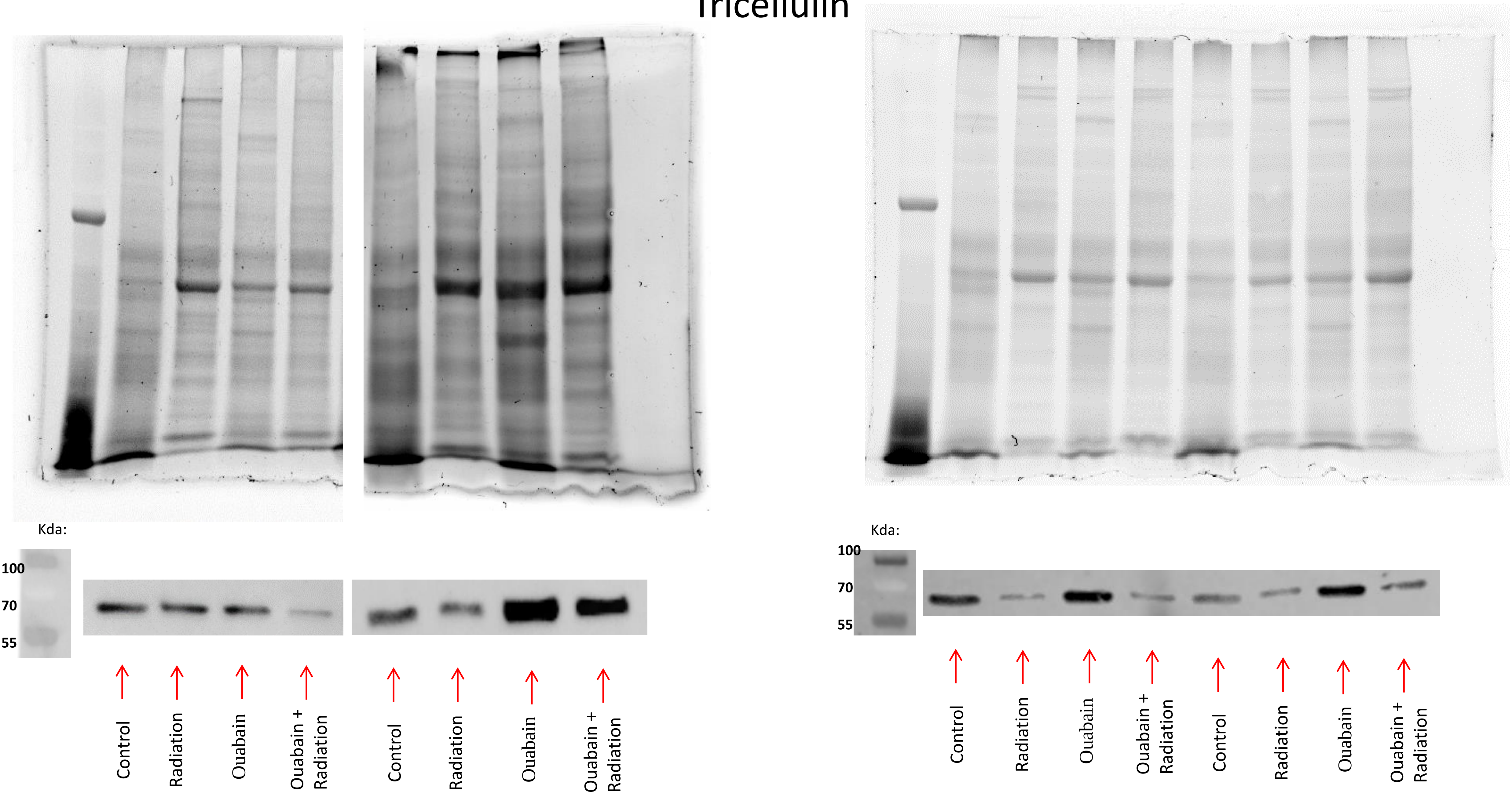

Activated caspase 3

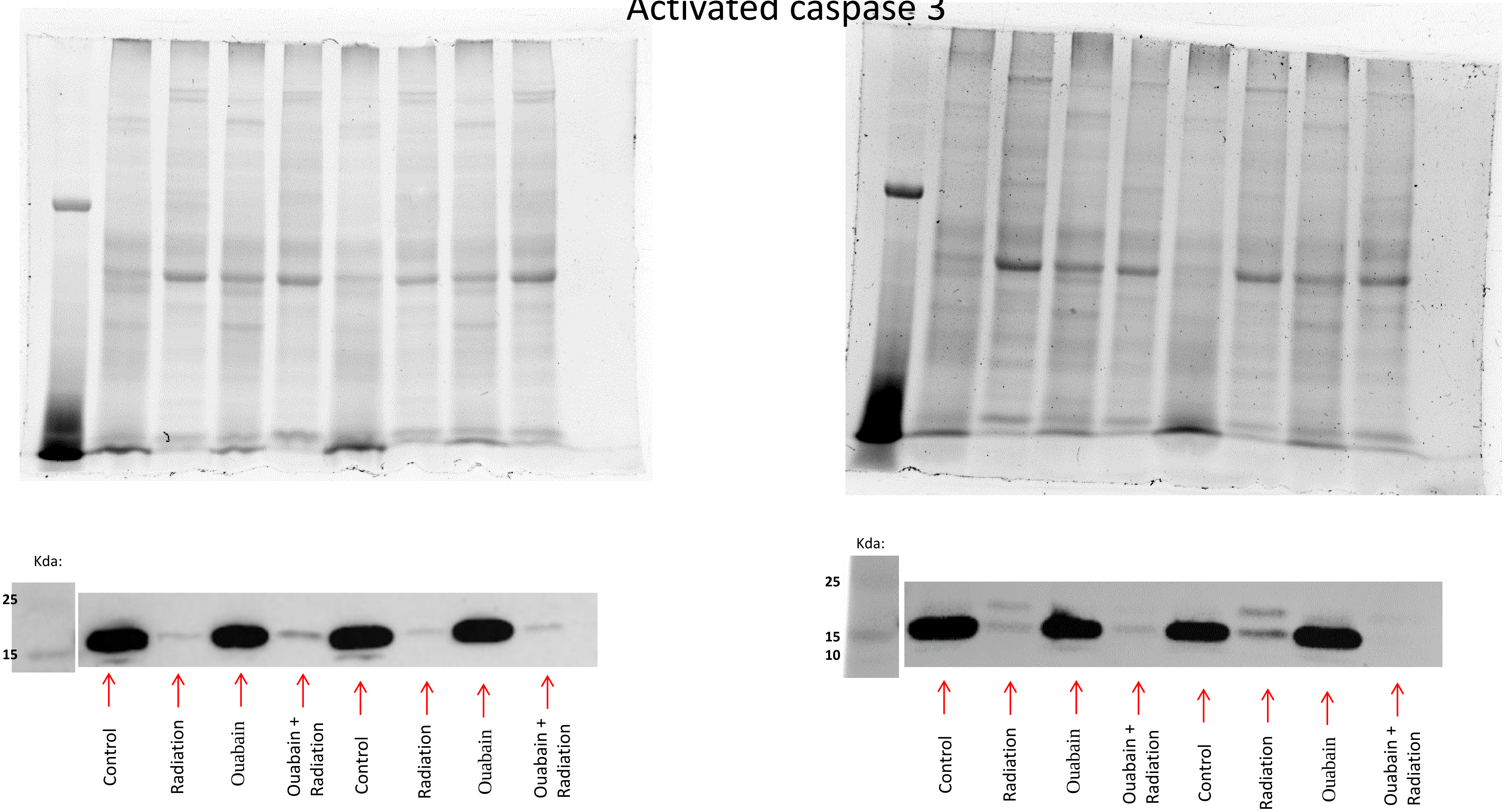

Colon

Claudin-1

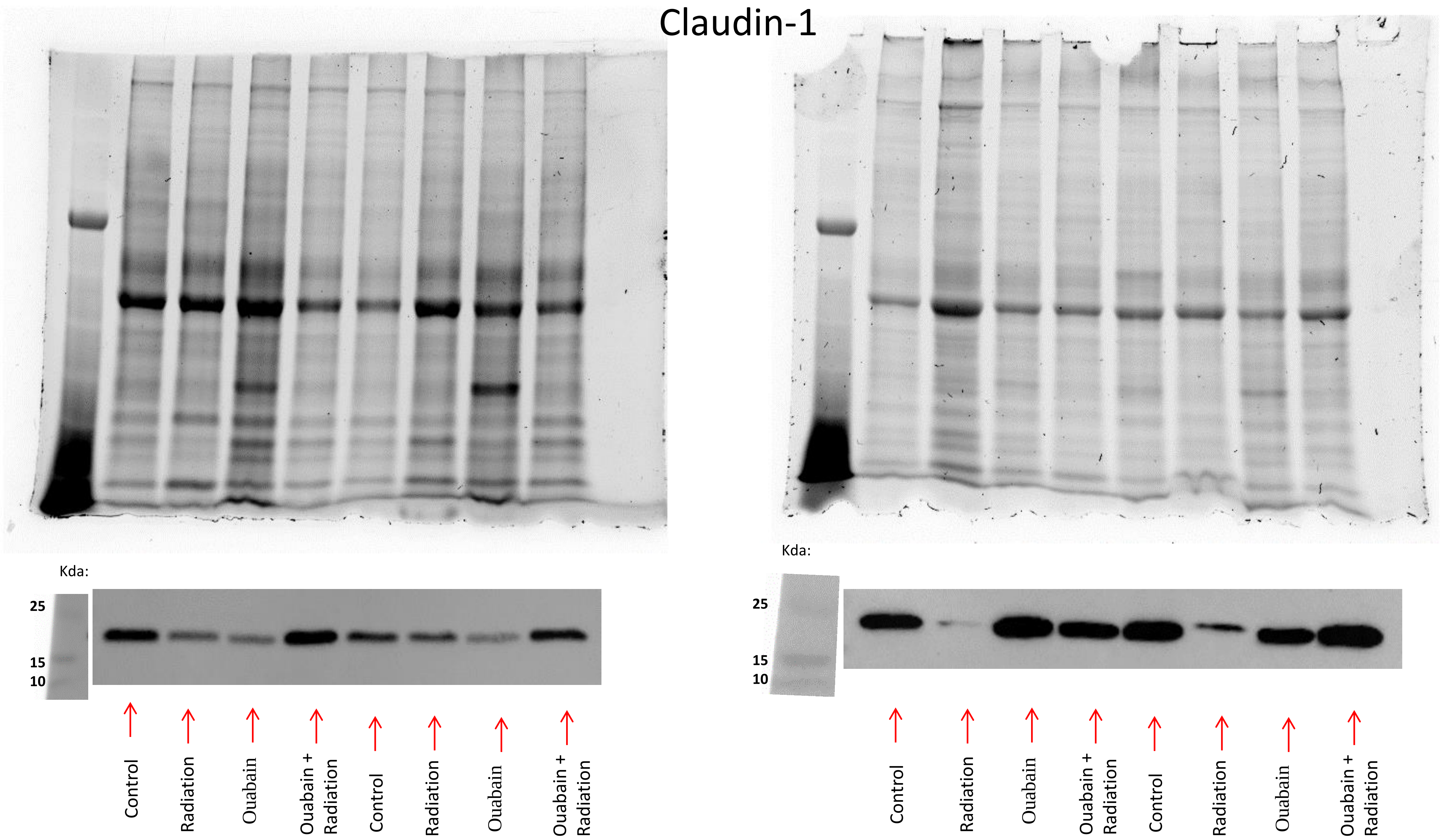
Kda:
25
15
10
Control
Radiation
Ouabain
Ouabain + Radiation
Control
Radiation
Ouabain
Ouabain + Radiation
Kda:
25
15
10
Control
Radiation
Ouabain
Ouabain + Radiation
Control
Radiation
Ouabain
Ouabain + Radiation

Claudin-2

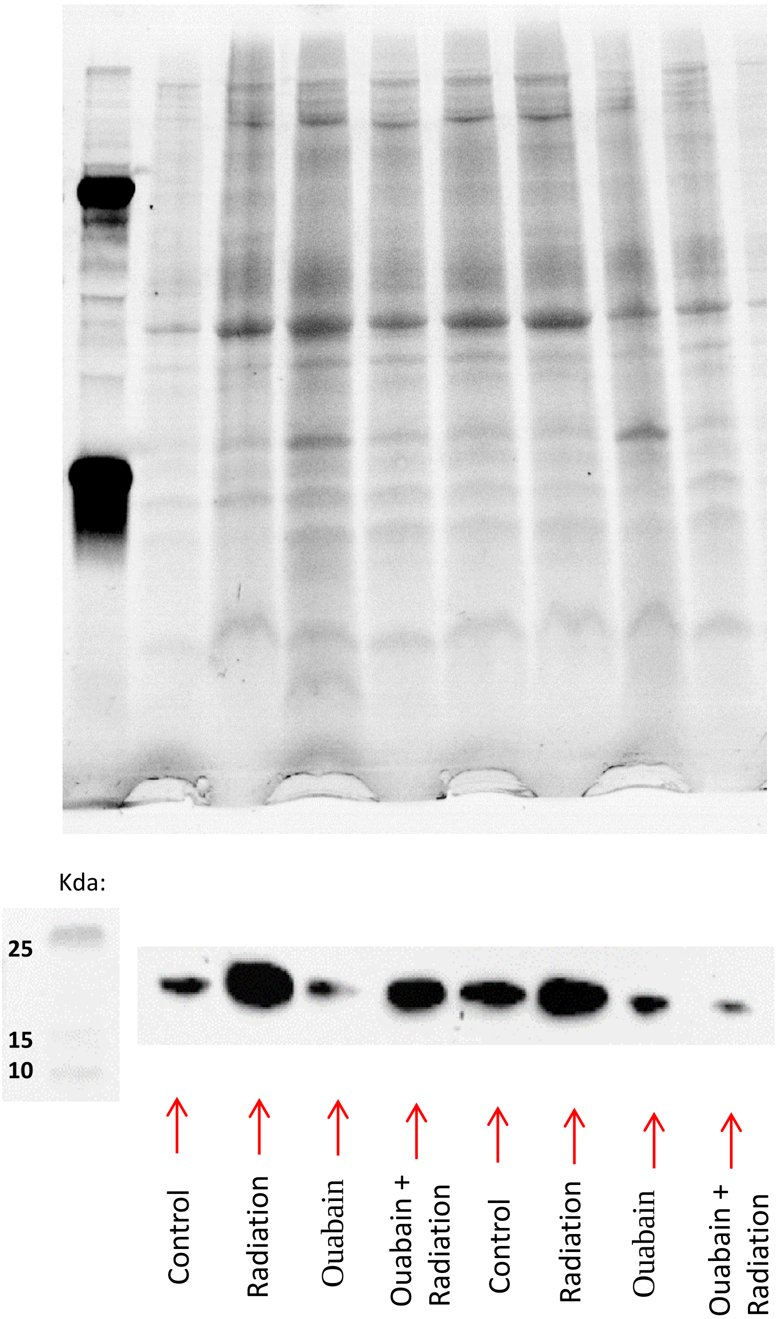
Kda:
25
15
10
Control
Radiation
Ouabain
Ouabain + Radiation
Control
Radiation
Ouabain
Ouabain + Radiation

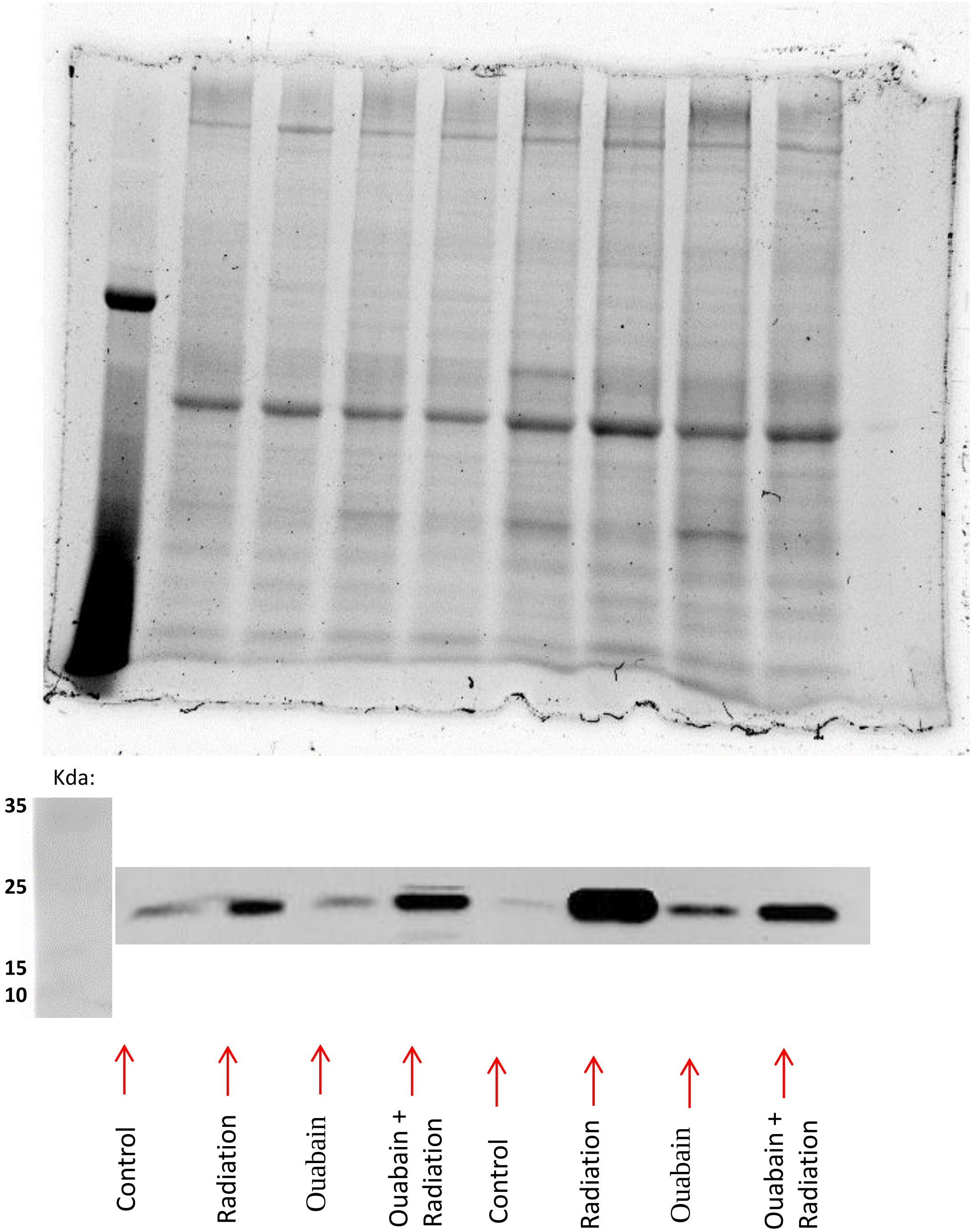
Kda:
35
25
15
10
Control
Radiation
Ouabain
Ouabain + Radiation
Control
Radiation
Ouabain
Ouabain + Radiation

Claudin-3

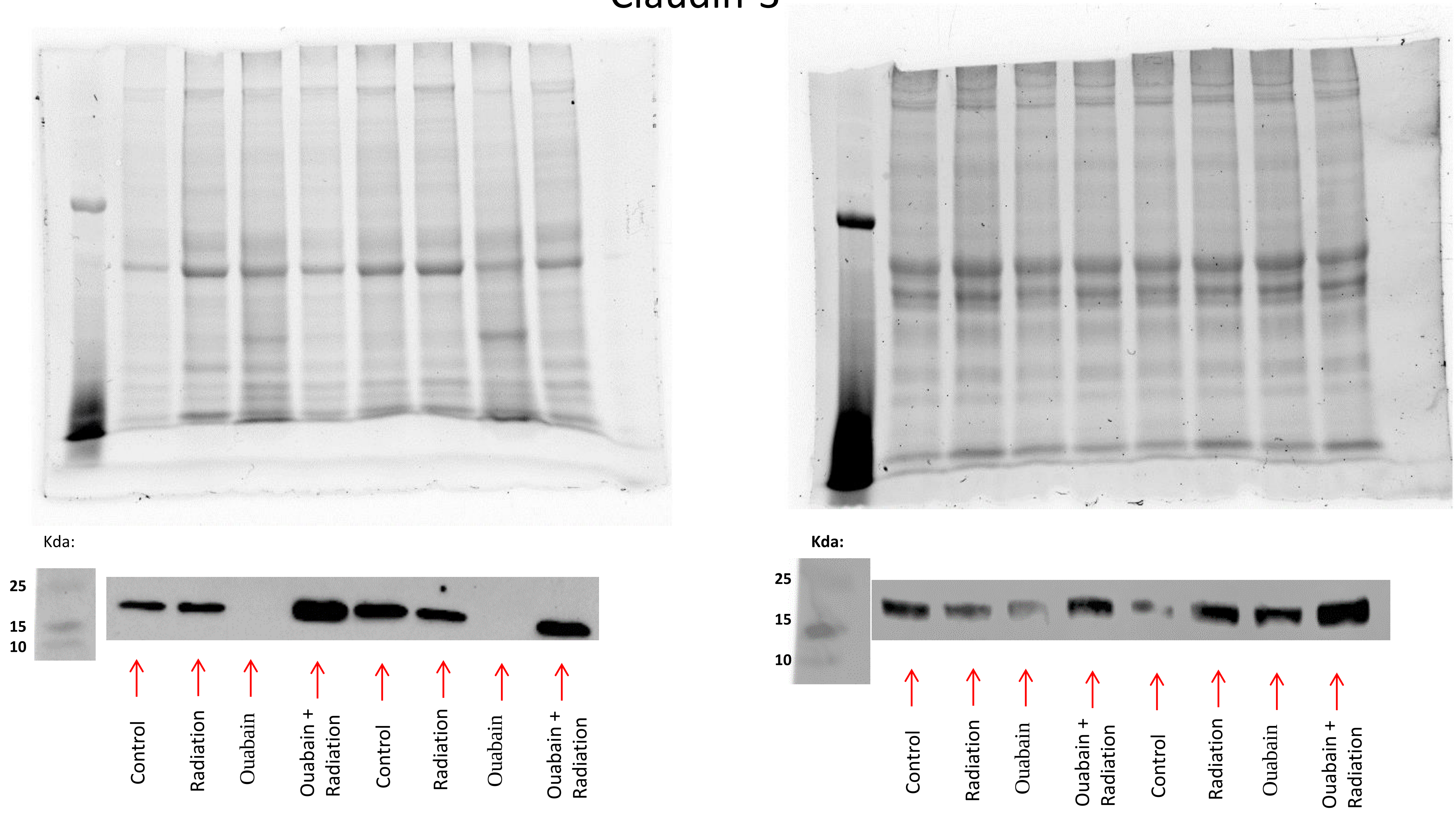
Kda:
25
15
10
Control
Radiation
Ouabain
Ouabain + Radiation
Control
Radiation
Ouabain
Ouabain + Radiation
Kda:
25
15
10
Control
Radiation
Ouabain
Ouabain + Radiation
Control
Radiation
Ouabain
Ouabain + Radiation

Claudin-4

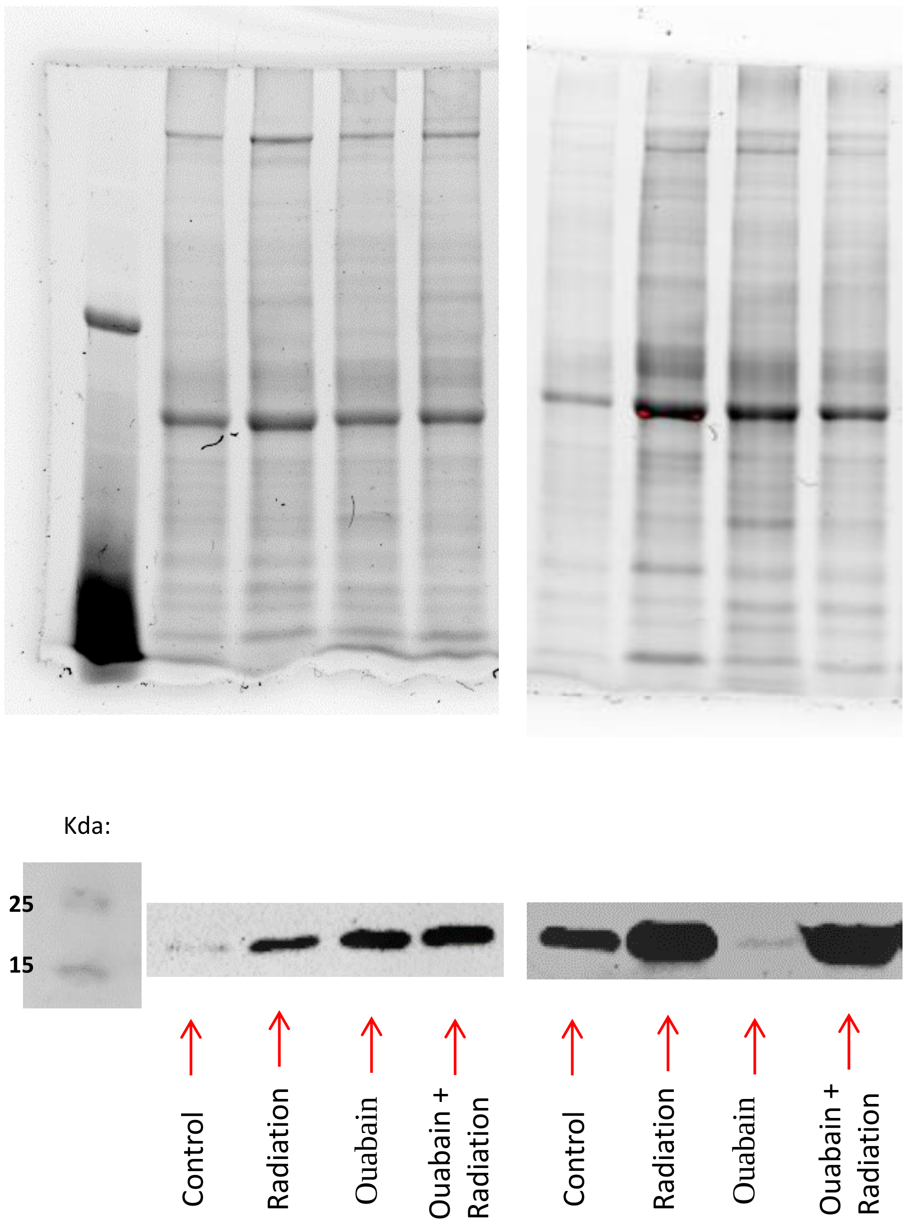
Kda:
25
15
Control
Radiation
Ouabain
Ouabain + Radiation
Control
Radiation
Ouabain
Ouabain + Radiation

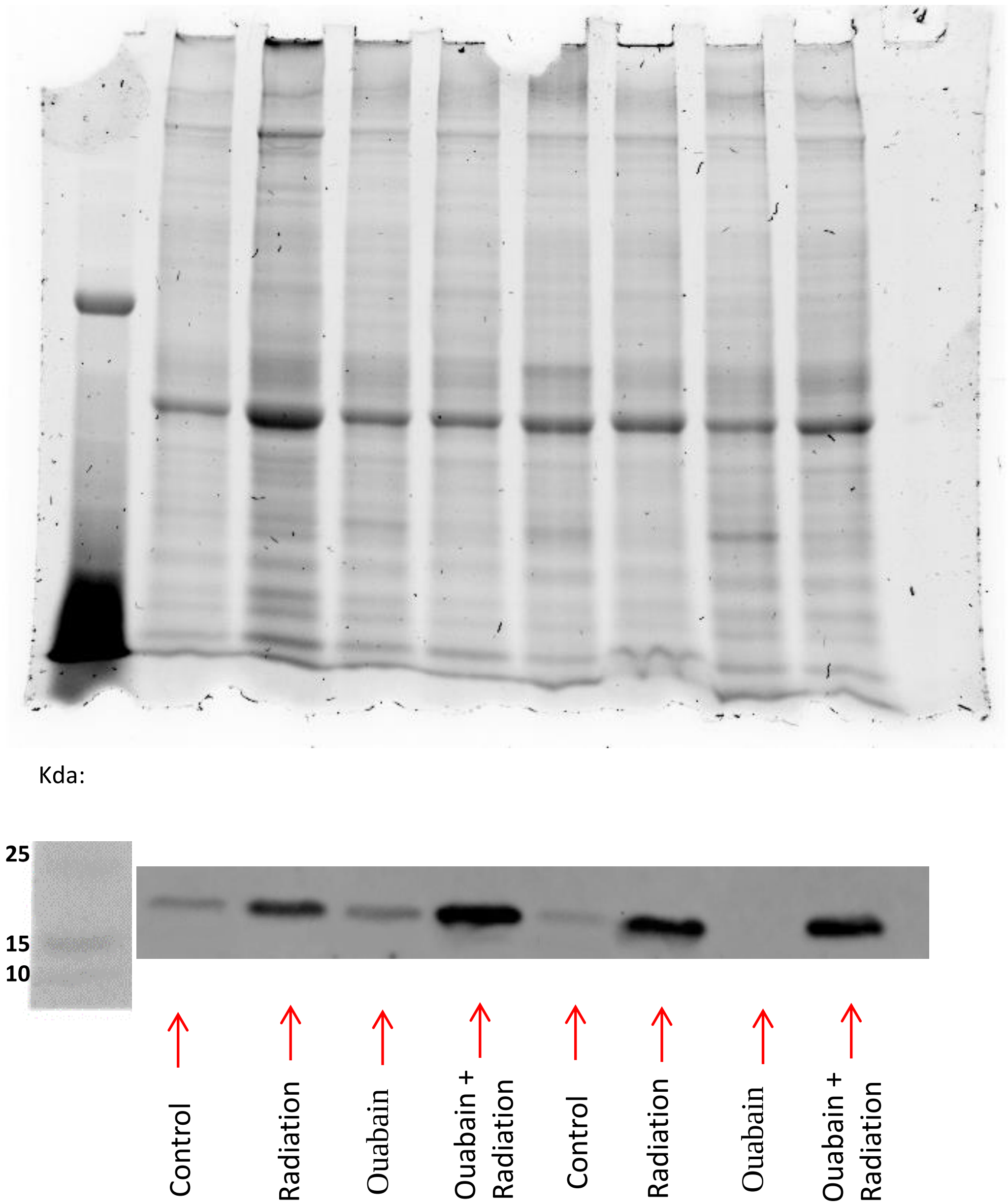
Kda:
25
15
10
Control
Radiation
Ouabain
Ouabain + Radiation
Control
Radiation
Ouabain
Ouabain + Radiation

Occludin

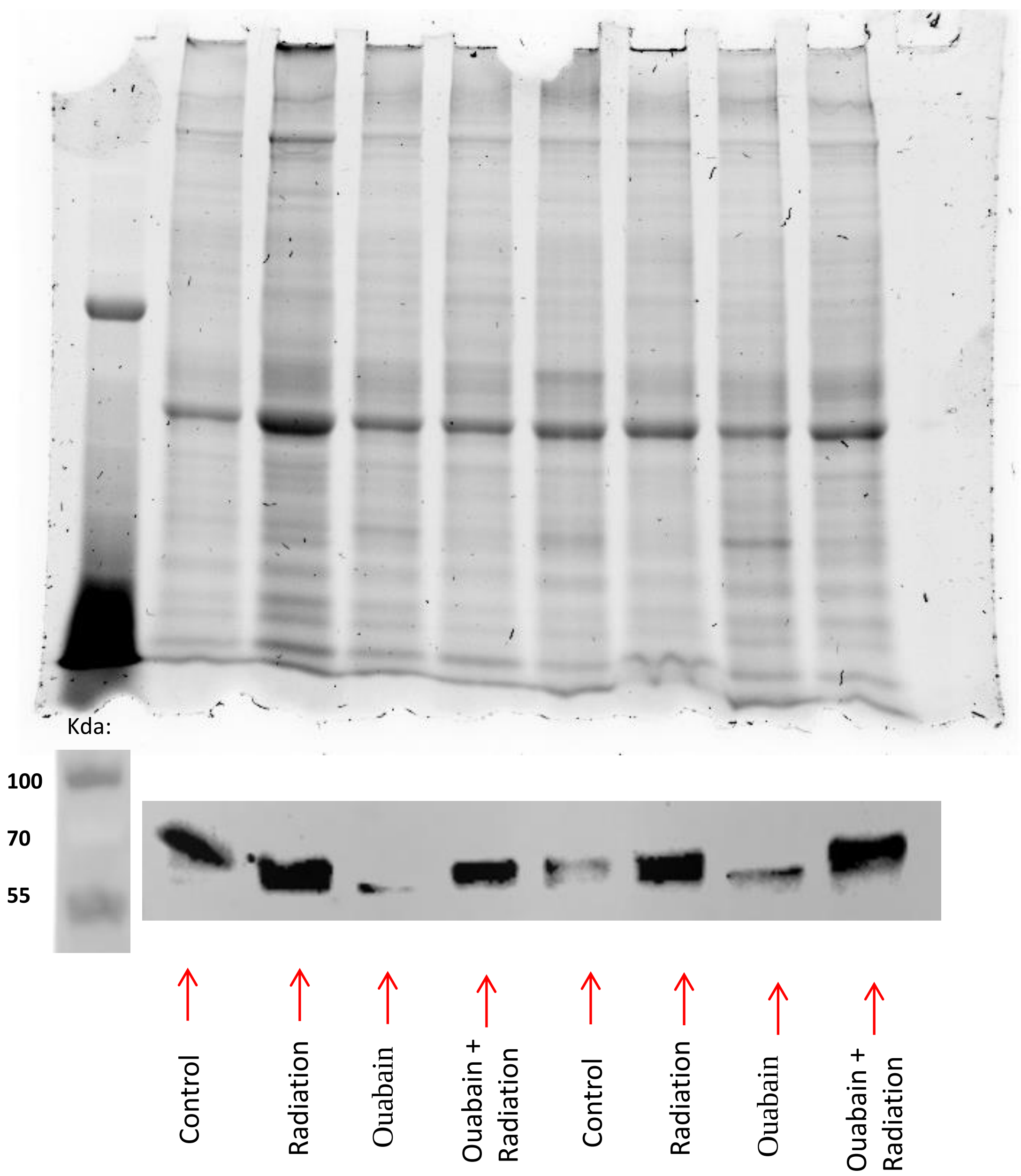
Kda:
100
70
55
Control
Radiation
Ouabain
Ouabain + Radiation
Control
Radiation
Ouabain
Ouabain + Radiation

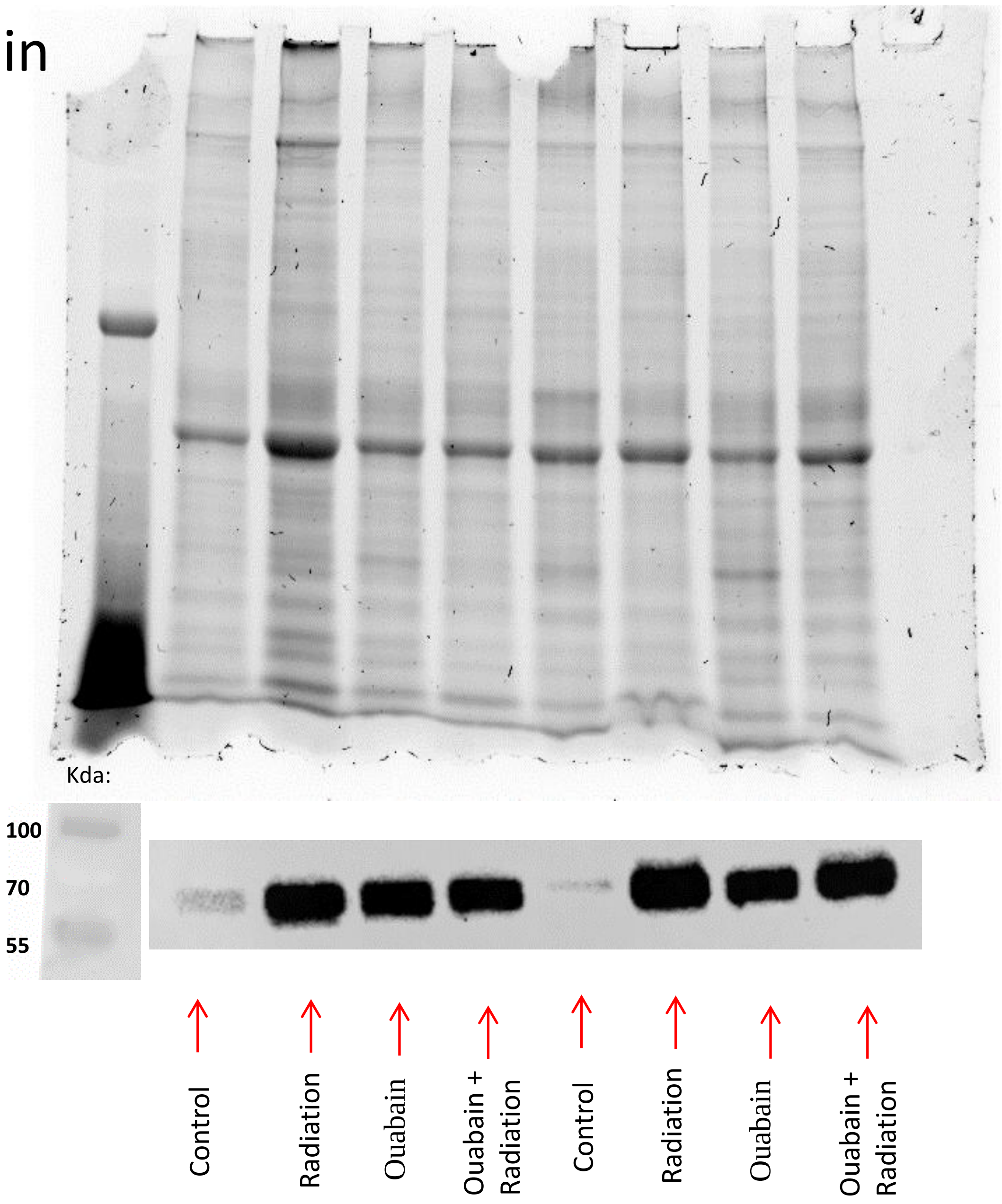
Kda:
100
70
55
Control
Radiation
Ouabain
Ouabain + Radiation
Control
Radiation
Ouabain
Ouabain + Radiation

Tricellulin

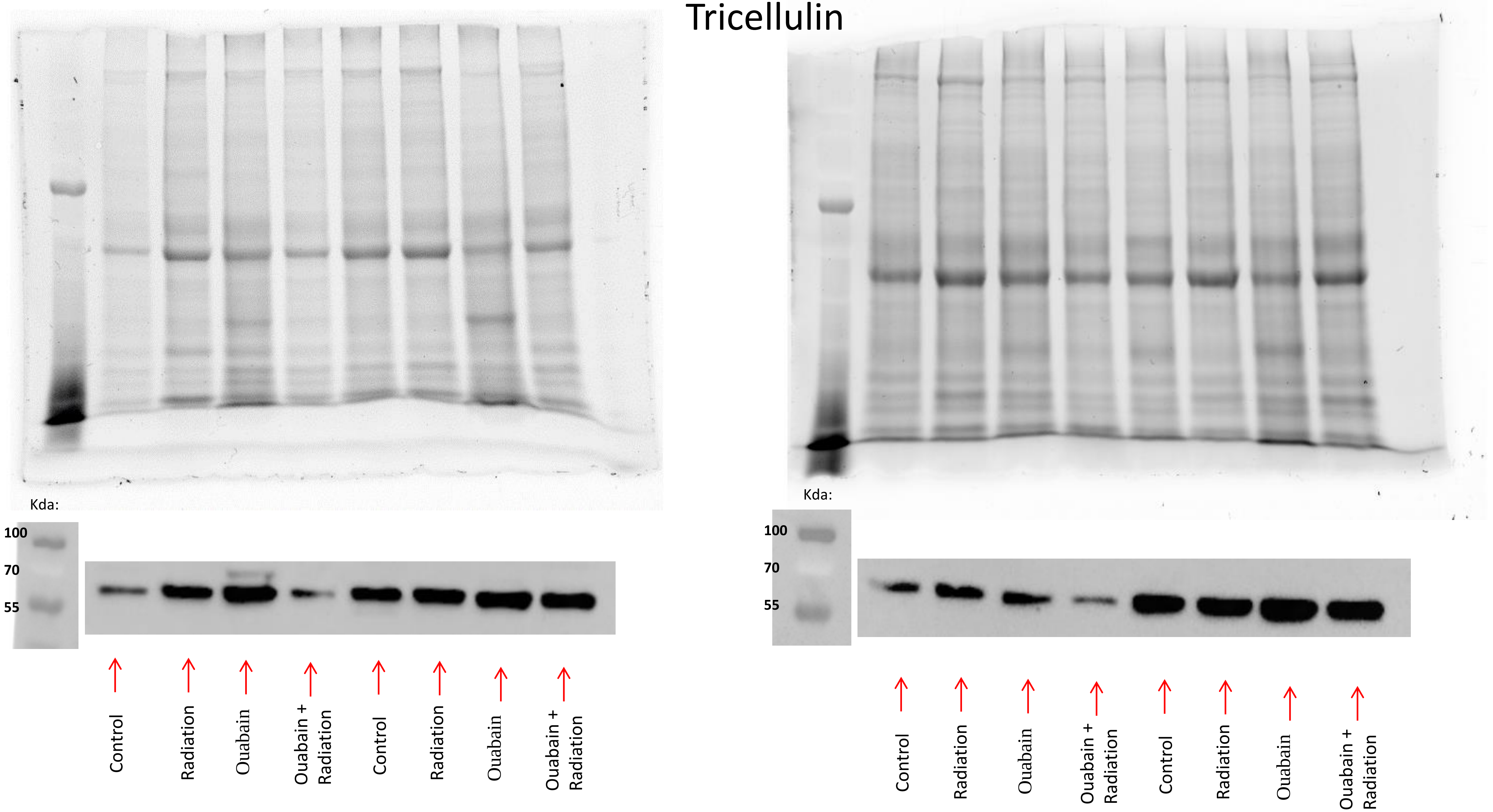

Activated caspase 3

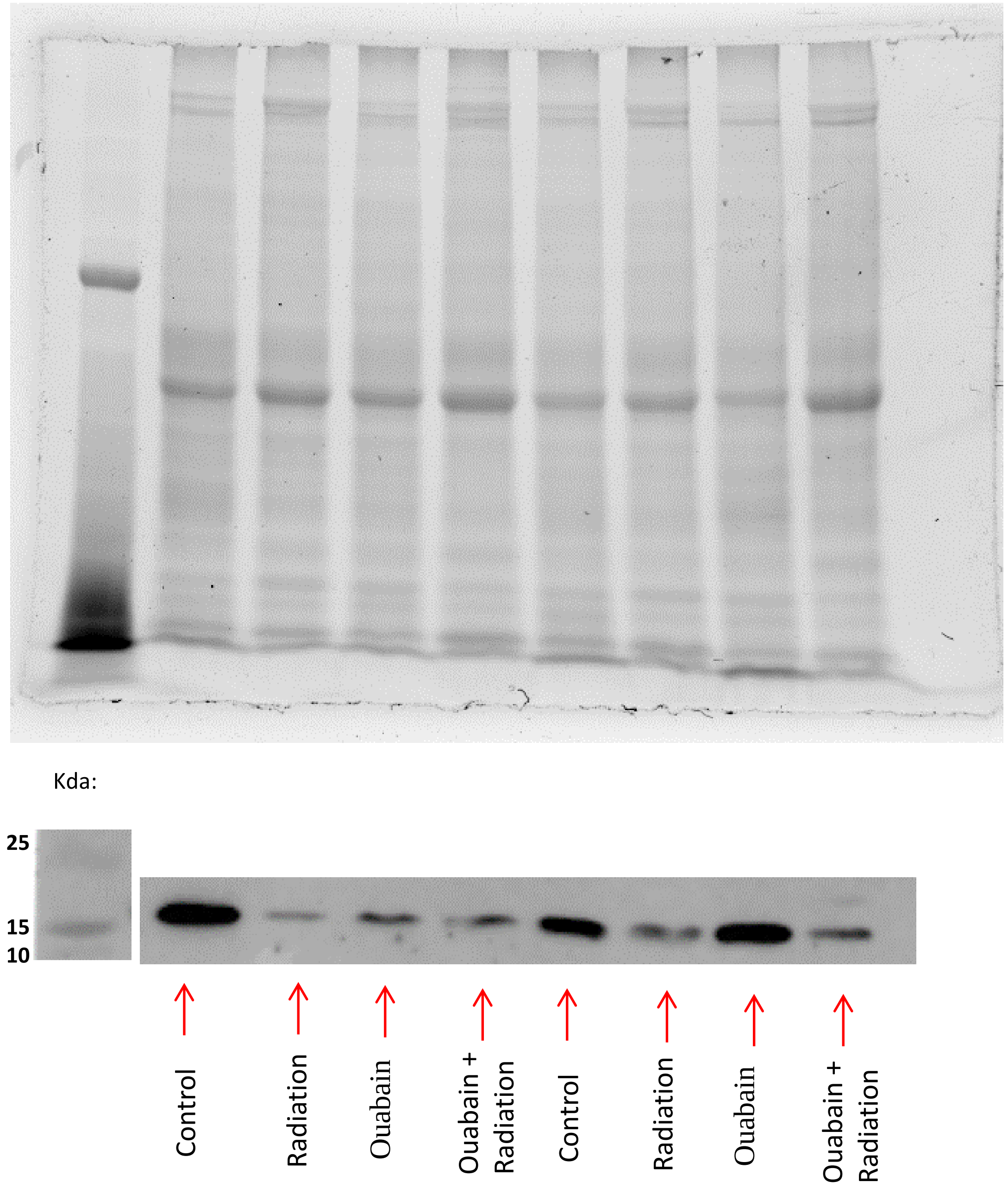

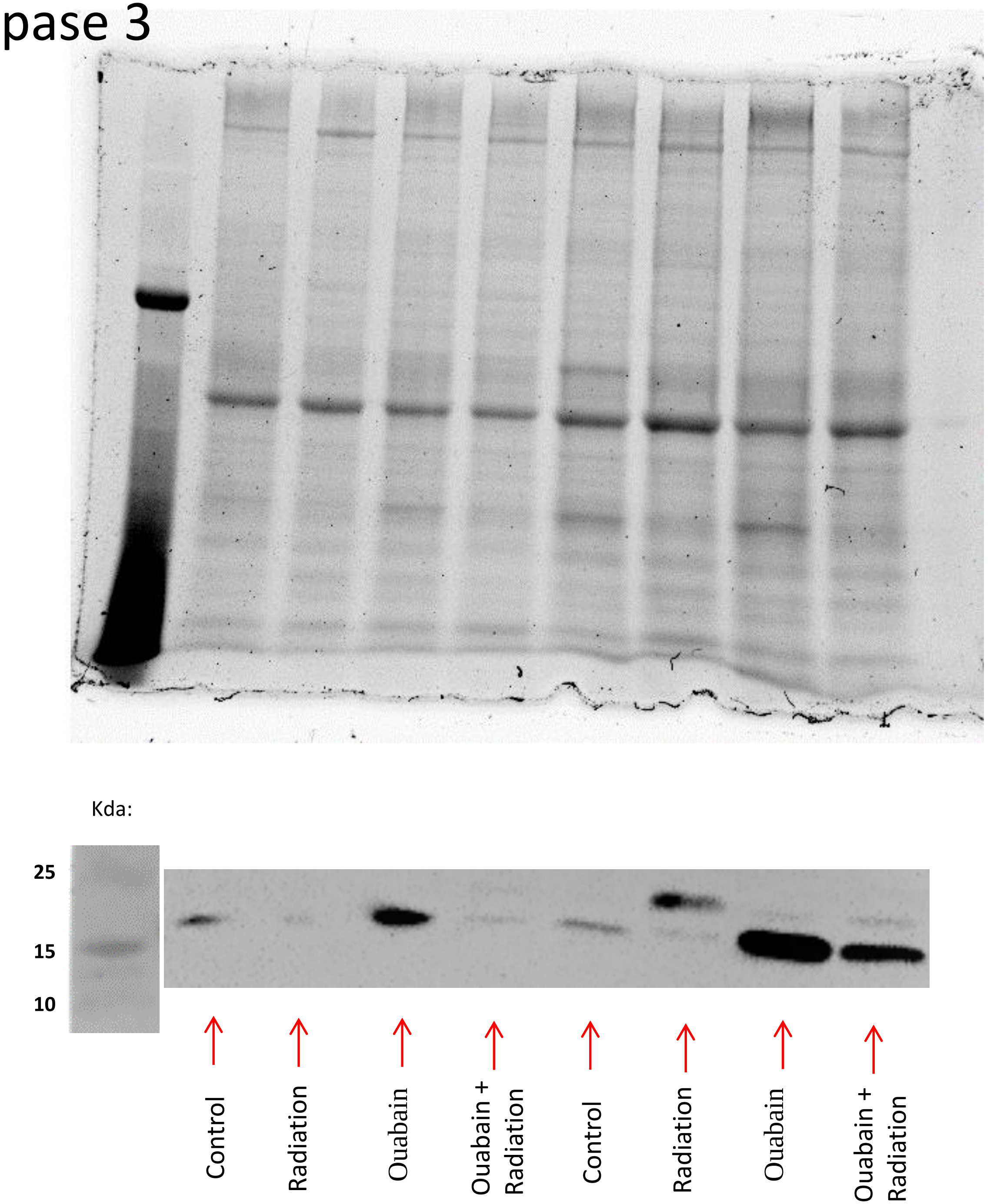

Supplement: Supplementary file 1 [file ijms-25-00278-s001.zip › ijms-2729024-supplementary.pdf]
